# Team communication patterns in emergency resuscitation: a mixed methods qualitative analysis

**DOI:** 10.1186/s12245-017-0149-4

**Published:** 2017-07-14

**Authors:** Lisa Anne Calder, George Mastoras, Mitra Rahimpour, Benjamin Sohmer, Brian Weitzman, A. Adam Cwinn, Tara Hobin, Avi Parush

**Affiliations:** 1 Department of Emergency Medicine, The Ottawa Hospital, Civic Campus, 1053 Carling Avenue, E-Main, Room EM-206, Box 227, Ottawa, Ontario K1Y 4E9 Canada; 2 Division of Cardiac Anesthesiology, University of Ottawa Heart Institute, 40 Ruskin Street, Ottawa, Ontario K1Y 4W7 Canada; 3 Clinical Epidemiology Program, Ottawa Hospital Research Institute, 1053 Carling Avenue, Room F6-58, Ottawa, Ontario K1Y 4E9 Canada; 4 Department of Psychology, Carleton University, Loeb B550, 1125 Colonel By Drive, Ottawa, Ontario K1S 5B6 Canada; 5 Department of Emergency Medicine, University of Ottawa, 1053 Carling Avenue, E-Main, Room EM-206, Box 227, Ottawa, ON K1Y 4E9 Canada

**Keywords:** Situational awareness, Resuscitation, Emergency department, Trauma, Cardiac arrest, Communication, Teams

## Abstract

**Background:**

In order to enhance patient safety during resuscitation of critically ill patients, we need to optimize team communication and enhance team situational awareness but little is known about resuscitation team communication patterns. The objective of this study is to understand how teams communicate during resuscitation; specifically to assess for a shared mental model (organized understanding of a team’s relationships) and information needs.

**Methods:**

We triangulated 3 methods to evaluate resuscitation team communication at a tertiary care academic trauma center: (1) interviews; (2) simulated resuscitation observations; (3) live resuscitation observations. We interviewed 18 resuscitation team members about shared mental models, roles and goals of team members and procedural expectations. We observed 30 simulated resuscitation video recordings and documented the timing, source and destination of communication and the information category. We observed 12 live resuscitations in the emergency department and recorded baseline characteristics of the type of resuscitations, nature of teams present and type and content of information exchanges. The data were analyzed using a qualitative communication analysis method.

**Results:**

We found that resuscitation team members described a shared mental model. Respondents understood the roles and goals of each team member in order to provide rapid, efficient and life-saving care with an overall need for situational awareness. The information flow described in the interviews was reflected during the simulated and live resuscitations with the most responsible physician and charting nurse being central to team communication. We consolidated communicated information into six categories: (1) time; (2) patient status; (3) patient history; (4) interventions; (5) assistance and consultations; 6) team members present.

**Conclusions:**

Resuscitation team members expressed a shared mental model and prioritized situational awareness. Our findings support a need for cognitive aids to enhance team communication during resuscitations.

**Electronic supplementary material:**

The online version of this article (doi:10.1186/s12245-017-0149-4) contains supplementary material, which is available to authorized users.

## Background

### Problem identification

Effective team communication is crucial in the resuscitation of critically ill patients. A team has been defined as two or more people who interact dynamically, have a common goal, a specific task and possess complementary skills [1]. During resuscitations, the team member composition may rotate in and out, members may or may not know each other’s roles and skills, and they may have varying tasks during the sequence of the event. Furthermore, these tasks are often time-critical and require the sharing of key pieces of data. Finally, an overall awareness of what is going on around you, or team situational awareness, is integral to successful resuscitation [2]. While the goal of successful resuscitation is restoration of circulation and an optimal clinical outcome, patient safety can at times be compromised resulting in adverse events.

When analyzing the root cause of adverse events, team communication has been a frequent theme [3, 4]. Recently, many efforts have been devoted to enhancing healthcare team performance via programs such as TeamSTEPPS, MedTEAMS and the practicing of non-technical skills via hi-fidelity simulation [5–10]. These efforts largely arose from a recommendation by the Institute of Medicine to use simulation focusing on teamwork with a view to enhancing patient safety [11]. One priority area of focus is helping teams achieve better situational awareness [2]. In order to build and maintain situational awareness, teams need to effectively communicate [12]. This can be challenging when resuscitation team members have little familiarity with each other and need to make rapid lifesaving decisions [13]. Resuscitations can be chaotic at times and situational awareness may be lost. In order to optimize team communication and enhance team situational awareness, we need to first understand resuscitation team communication patterns. This has yet to be fully described in the literature.

### Study goals and objectives

The overall goal of this study was to understand how teams communicate during resuscitation, whether they endorsed a shared mental model and their information needs. We defined a shared mental model as an organizing knowledge structure of the relationships among the team [14]. Specifically, our objectives were to (1) gain a better understanding of shared mental models of resuscitation team members; (2) determine communication patterns in terms of types of communication, content of information exchange and types of team interactions; and (3) determine information needs for team situational awareness.

## Methods

### Study design and setting

This was a mixed methods observational study with three components: (1) stakeholder interviews; (2) observations of recorded simulated resuscitations; (3) live observations of resuscitations in the emergency department (ED). The study was conducted at The Ottawa Hospital, a tertiary care academic center and level 1 trauma center in Canada where the dual campus EDs have a census of 150,000 ED visits per year. The Ottawa Hospital Research Ethics Board approved the study.

### Study population

#### Interviews

We engaged in purposive sampling in order to represent all potential professions of stakeholders in resuscitation teams in an ED setting. These included attending physicians, trauma team leaders, emergency medicine residents, registered nurses, respiratory therapists, advanced care paramedics, patient care assistants, registration clerks, and social workers. We excluded medical students who do not tend to take an active role in resuscitations, but rather serve as observers. We also excluded patients who are often too ill to be aware of resuscitation procedures.

#### Simulation observations

We included all emergency medicine residents and registered nurses who provided informed consent to have their simulated resuscitations recorded and analyzed.

#### Live observations

We included any or all of attending emergency physicians and residents, consultant physicians, registered nurses, respiratory therapists, administrative clerks, patient care assistants, paramedical personnel and social workers. We included resuscitations for patients suffering from shock states (septic, hypovolemic, cardiogenic, obstructive, anaphylactic, neurogenic), cardiac arrests (both “Vital Signs Absent” and “Return of Spontaneous Circulation” cases), other unstable cardiac dysrhythmias, trauma resuscitations meeting institutional trauma team activation criteria, and other illnesses requiring an overhead Emergency Physician “stat call” as dictated by nursing discretion. Given the emergency nature of the care provided and the de-identified methods of data collection, the research ethics board waived the requirement of informed consent for this component of the study.

We excluded cases involving patients less than 18 years of age as these are rare and not typical at our center. We also excluded cases where resuscitation is not as intensive, often requiring a smaller team: patients with stroke codes, respiratory distress not requiring intubation (e.g., COPD exacerbation, patients placed on non-invasive ventilation), uncomplicated acute heart failure, stable agitated patients, trauma not deemed to require trauma team activation. We also excluded transfers from outside hospitals directly to the care of admitting services.

### Data collection

#### Interviews

Our review of the literature could not uncover any survey tools designed to assess shared mental models of resuscitation teams. We designed the interview questionnaire using psychology and clinical resuscitation expertise of the investigative team. Questions were designed to determine team member roles and responsibilities, procedural expectations, and devices and artifacts used in resuscitation. The interview questionnaire is presented in Additional file 1: Appendix A.

The interviews were semi-structured and conducted by two of three investigators (AP, MR, TH) in a hospital conference room. Interviewees were provided with an information sheet detailing objectives and nature of the study and written informed consent was obtained. Notes were taken and all interviews were audio recorded. Interviewers used pre-defined prompts, probes, and follow-up questions.

#### Simulation observations

We studied regularly scheduled critical care simulations which were part of the existing curriculum for those University of Ottawa Emergency Medicine residents who consented to participate in the study. Each simulation involved one to three residents at the post-graduate 1–5 year levels and 1–3 registered nurses. Emergency Medicine residents would also act as consultants in the simulations. Simulations were recorded with 3 separate cameras by the University of Ottawa Skills and Simulation Center for the purpose of education and delivering feedback during simulation debriefing sessions. Each group of videos were packaged together using Owl video player software. The video player allowed the viewer to observe four videos in four different windows at the same time. The videos were viewed and transcribed by a single investigator (MR). The transcriptions were entered into separate worksheets for each video, using Numbers and Microsoft Excel 2010 in four categories: (a) time and duration; (b) source and destination; (c) information conveyed; and (d) action. Verification of accuracy of data entry was performed by a clinician investigator (GM) for 10% of cases. The videos were permanently deleted at the end of the study.

#### Live observations

We prospectively observed a convenience sample of ED resuscitations based on the availability of a clinician investigator (GM). The investigator did not participate in the observed resuscitations. Observations proceeded from activation of the resuscitation team (e.g., by Emergency Medical Services (EMS) patch, overhead “stat call”, or initiation of overhead “code” calls) until patients were deemed stable or deceased by the most responsible physician (MRP), or care was handed off to consulting services.

We captured all instances of communication between team members in written notes using a structured and piloted data collection form based on concepts in other health-care communication research [15, 16]. We recorded baseline characteristics of the resuscitations including patient age and gender; length of resuscitation before termination of the observation (in minutes); number and roles of participants involved in the resuscitation; and patient disposition and outcome at the time of leaving the ED (e.g., home, intensive care, operating room). Nursing records from each resuscitation were de-identified, photocopied, and used after observation to cross-reference and verify the collected data. We entered data from observed resuscitations into a spreadsheet database (Microsoft Excel, 2010). We estimated that we would require 20 observed cases to obtain data saturation, when no further new themes emerged, but were able to achieve this after observing 12 resuscitations.

### Outcomes measures

#### Interviews

Data were abstracted into a data collection form divided into five domains: (a) resuscitation event types; (b) flow and sequence of events: detailed explanation of a recent resuscitation; (c) people, tasks and information: team member interaction and linking for task completion; (d) problems, challenges or obstacles: any issues that could have led to communication breakdown; (e) communication network: list of team member interactions, events that could lead to such interactions. The outcome of the interviews was a perspective-based map of the workflow during resuscitations, including the role and responsibilities of each team member, expected coordination and information sharing processes.

#### Simulation and live observations

Using communication analysis we outlined and quantified verbal behaviors (e.g., questions, replies), content categories (e.g., patient status, medications), situation awareness communications, sequential diagrams of communications (frequencies of communication sequences between team members), and mapping all of the above as a function of the event timeline.

### Analysis

A qualitative communication content analysis method was used to identify, categorize, and aggregate items of information shared by the team during resuscitations to identify common thematic elements [17]. We triangulated these thematic groupings with data collected during the interviews, simulated and live observations and reconciled differences in classification to identify main thematic elements. Edge lists were created to analyze frequency of interactions between various providers in the resuscitation. Analysis proceeded in an additive fashion as we generated new observations. We performed a gap analysis between the expected team processes as reflected by the interviews and the actual processes captured in the simulator sessions. We expected gaps in expectations between team members to be reflected in team processes breakdowns.

## Results

### Interviews

**Fig. 1:**
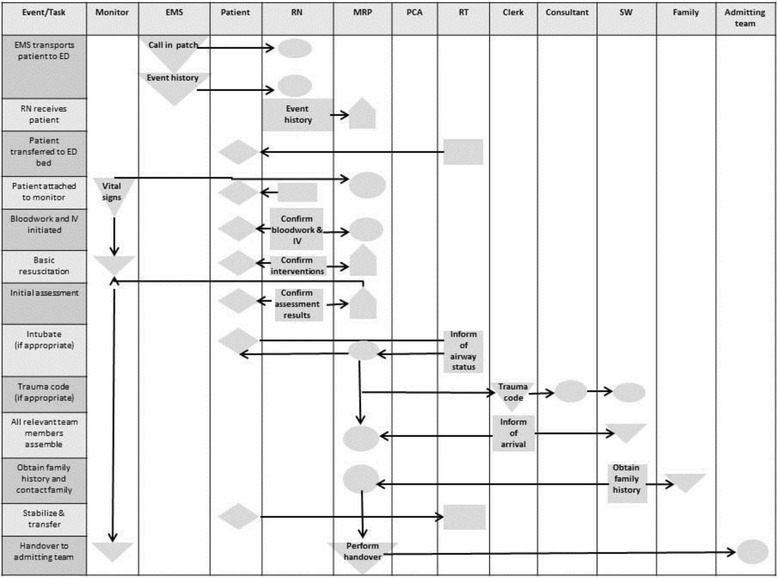
Swim lane representation of event sequence flow for resuscitation from 18 interviews

**Fig. 2:**
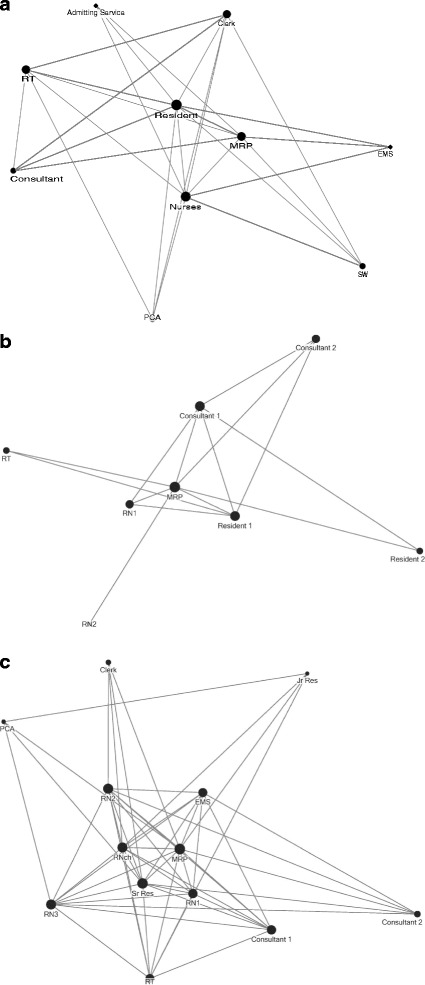
Social network representations of resuscitation team-based communication

We interviewed 18 stakeholders in resuscitation, nine women and nine men (Table 1). The range of years of experience with resuscitations was from 2 to 30. We found that interviewees described potential resuscitation scenarios which clustered into 6 types: trauma, cardiac, overdose, major hemorrhage, sepsis and respiratory failure. We asked respondents to describe a typical flow sequence of events for resuscitations and from these created a swim lane representation (Fig. 1). Initially, information flows from EMS to the nursing team and MRP. Subsequently, communication can include the patient but most often is between the nurse and MRP. As the case evolves, other team members such as residents, respiratory therapists, administrative clerks, social workers communicate primarily with the MRP and/or a nurse. The swim lane figure demonstrates that the majority of the work and communication flow is via the MRP and the nurse, where the MRP holds the majority of the interactions and decision making and the nurse the majority of the actions. Figure 2a depicts the social network patterns of communication derived from the interviews.

**Table 1:** Roles and goals of resuscitation team members described during 18 interviews

Role, N	Goals
Attending emergency physician, 4	• Provide the best care in a timely organized manner that is safe for the patient • Effectively resuscitate a patient and either deliver definitive care or delineate what definitive care is required • Overall patient survival, identifying injuries needing treatment urgently, identifying what services need to be involved, what needs to be done now—prioritizing what treatments are needed, what services needed to be involved and what investigations need to be done
Trauma team leader, 2	• Keep patient alive, treat any presenting injury in the best way possible, as quickly as possible • “global vision” of the resuscitation scenario • Take the injured patient, resuscitate them, identify all their injuries and make sure that all of their injuries are dealt with in a timely manner • Coordinate everyone else, all bodily systems get dealt with
Charting nurse, 1	• Have a complete oversight of the whole resuscitation situation, ensuring that all critical information, times, interventions, medication administrations and specialist involvement are documented for patient and hospital records
Task nurse (intravenous access), 1	• Efficiently and effectively gain IV access on the patient upon arrival and obtain, send and receive laboratory work and results expediently
Task nurse (monitor), 1	• Efficiently and effectively hook up the patient to monitors upon arrival • Taking care of the patient and continuing care ensuring there is follow up after blood results • Give proper treatment • Foresee if the patient is deteriorating
Emergency medicine resident, 2	• Participate in the treatment and management of the patient to obtain further experience in emergency situations
Respiratory therapist, 2	• To help the patient achieve a clear airway
Administrative clerk, 2	• Quickly and efficiently page for or locate any supplies, services or devices required for the physicians and nurses to effectively manage and treat the patient
Patient care assistant, 1	• Quickly and efficiently moving or transferring the patient to appropriate location, being “hyper aware” for any missed injuries or contusions on the patient’s body
Social worker, 1	• Working in parallel alongside the resuscitation team as both the patient’s and family member’s advocate, being the family members eyes and ears in the trauma situation. • Attend to the family’s needs in the most efficient and effective way possible
Paramedic, 1	• Safely transport the patient to the care of the hospital with all the information that has directed care up until that point and will continue to direct care when the hospital team takes over

The interviewed stakeholders described their roles and goals in resuscitation (Table 1). The goals identify needs for overall vision of the scenario, immediate life-saving actions, coordination of care, working rapidly and efficiently as well as anticipation of adverse events. When asked about problems in team communication during resuscitation, respondents described three types: communication, organizational and environmental. Communication problems included errors of omission or failure to communicate vital information: “…a lot of physicians do not call out results of their assessments to the charting nurse”; “individuals entering the resuscitation not announcing who they are when they show up”; “no set protocol in place for information which should always be communicated directly to the team leader”. Organizational problems were related to policy, protocol or procedure: “not receiving the ambulance call record”; “do not resuscitate wishes not known”; “interventions that were done not documented”. Environmental factors were described as issues with the resuscitation environment: “too much noise”; “too many people”; “too much distraction”.

### Simulation observations

We observed 30 simulated resuscitations, involving a total of 112 residents and nurses (Table 2). We observed a total of 2625 information exchanges over a mean case duration of 13 min. Figure 2b demonstrates the social network representation of the teams in simulation. We found the following types of communication behaviours sorted by their frequency: statements (27.2%), directives (23.6%), questions (17.1%), acknowledgements (13.8%), clarifications (10.1%), (2.7%), explanations (1.9%), instructions (1.4%), and suggestions (0.8%). Of these, statements, questions, clarifications, explanations are most relevant to situational awareness. The information content categories are summarized in Table 4.

**Table 2:** Characteristics of 30 observed simulated resuscitations

Type of case	Number of simulated resuscitations	Number of participants (median, range)	Duration (min) of simulation (median, range)
Cardiac arrest	21	3, 3–7	11.5, 4–28
Trauma	4	5, 3–8	16, 4–29
Sepsis	1	4, –	14, –
Status epilepticus	2	3, 3–3	10.5, 9–12
Respiratory failure	1	4, –	16, –
Anaphylaxis	1	5, –	9, –

### Live observations

We observed 12 live resuscitations involving a median of 10 team members per case (Table 3). We documented a total of 2,128 information exchanges during a mean case duration of 24 min. Social network analysis (Fig. 2c) demonstrates that the MRP, recording nurse, and senior resident were the most central figures within the team, involved in 24.5, 17.3, and 16.4% of all communications respectively. Communications involving the bedside nurses comprised another 21.8% of all communications. We categorized the types of communication exchanges and found, in order of frequency: statements (18.9%), requests (18.3%), questions (17.7%), answers (14.1%), replies (10.6%), broadcasts (5.6%), acknowledgements (5.2%), clarifications (3.5%), and read backs (2.9%). The information content categories are summarized in Table 4, Additional file 1: Appendices B and C. Examples of information exchanges are provided in Additional file 1: Appendix D.

**Table 3:** Characteristics of 12 observed live resuscitations

Type of case	Number of resuscitation cases	Patient age (median, range)	Patient sex, male (*n*)	Number of ED resuscitation team members (median, range)	Duration (min) of observed resuscitation (median, range)	Outcomes			
						Death	ICU/CCU*	OR**/cardiac catheterization	Ward
Cardiac arrest	5	73, 56–84	5	10.0, 7–14	17, 6–32	2	1	2	0
Trauma	3	36, 18–68	2	12.0, 11–13	13, 13–32	0	0	1	2
Sepsis	2	86, 78–93	1	8.5, 7–10	52.5, 47–58	0	2	0	0
Status epilepticus	1	42, –	0	6	15	0	0	0	1
Respiratory failure	1	54, –	1	9	26	0	1	0	0

*ICU* intensive care unit, *CCU* coronary care unit, *OR* operating room

**Table 4:** Key information category themes from each method

Interviews (*n* = 18)	Simulated observations (*n* = 30)	Live observations (*n* = 12)	Final consensus categories
Time	Time (included time since last epinephrine, duration of cardiopulmonary resuscitation and “cycling” or frequency of blood pressure monitoring)	Resuscitation status (patient status and time elapsed)	Time
Vital signs	Patient status^a^	Vital Signs	Patient status^a^
Patient assessment	History (included allergy status, “down time” and mechanism of injury)	History	History
Medications	Medications	Treatments	Interventions^b^
Investigations	Investigations (included x-ray, computed tomography and bloodwork)	Investigations	Assistance and consultations
Interventions	Interventions	Clinical findings	Team members present
Team members present	Treatments	Intravenous access	
	Assistance requests and consultations	Equipment	
	Codes (included activation of the trauma team, cardiac catheterization lab and security team for violent patients)		

^a^Includes vital signs

^b^Includes medications, investigations, treatment

### Gap analysis

The social network analysis for the interviews (Fig. 2a) demonstrates the expected communication patterns during resuscitation. The majority of communications occurred between the MRP, nursing team and consultant with the respiratory therapist, clerk and patient care assistant more peripheral in their involvement. The recorded simulation observations (Fig. 2b) show a more simplified network; however, there were fewer participants in these simulations and not all roles from the interviews were represented. Despite this, the theme of majority of communication between MRP and nurse is preserved. The live observation social network (Fig. 2c) reflects even greater complexity than expected with more involvement of EMS but more peripheral involvement of respiratory therapist, clerk and patient care assistant as expected. Comparison of the key information categories (Table 4) shows that all expected categories from the interviews were represented in the observations with the exception of team members present which is a non-verbal, visual piece of information.

## Discussion

### Key findings

We found that resuscitation team members have a shared mental model which involved understanding each other’s roles and goals in the resuscitation in terms of providing rapid, efficient, life-saving care with a need for overall situational awareness. The problems identified by stakeholders reflected environmental factors (excess noise, distraction) and potential information loss in both verbal and written communication. The flow of information described in the interviews was reflected by the observations of simulated and live resuscitations. The most responsible physician and charting nurse were central in terms of volume of communication and the most common types of communication involved statements, requests, questioning and acknowledging. These types of communication were brief and direct rather than less frequent communication types such as suggestions and explanations which fits the need for rapid and efficient communication. These patterns also reflect the communication activity that is required for the acquisition and maintenance of situational awareness as well as teamwork processes such as coordination and cooperation. The categories of data conveyed during resuscitations were quite consistent among all 3 methods and we consolidated these into (1) time; (2) patient status; (3) patient history; (4) interventions; (5) assistance and consultations; (6) team members present. These elements, in addition to verbal behaviours such as statements and questions are all fundamental to establishing and maintaining situational awareness during resuscitation as they include information about the patient, environment, task, and time [2].

### Context with existing literature

Our findings are consistent with a previously described integrative framework of task-related teamwork behaviours since we observed communication patterns consistent with team coordination, cooperation and information exchange [18]. In terms of previous empiric observational research on resuscitation teams, investigators have focused on specific aspects of team functioning such as leadership, adherence to existing protocols and guidelines as well as communication patterns between resuscitation physicians [12, 19–21]. Others have examined the shared mental model of resuscitation teams via survey or observation of pediatric trauma management [22, 23]. Our study confirms that multiple collaborators are involved in information sharing during resuscitation. Our findings contrast with previous work since we found that team members did in fact have a shared mental model in our interviews and we identified the recording nurse as central to resuscitation team communication. Our study is the first comprehensive mixed method investigation of how inter-professional teams communicate during ED resuscitation.

### Research implications

We found that resuscitation team members share large volumes of critical information throughout a resuscitation event. A large proportion of communication is oriented around confirmation of clinical findings and patient status. We noted a high volume of communication between the MRP and charting nurse, reinforcing the shared mental model described in the interviews. Given the time constraints of these team communications, it is easy to appreciate the risk of information loss and degradation of situational awareness. This presents a potential threat to patient safety.

Future research needs to consider solutions to enhance the acquisition of key information by all team members, maintenance of team situational awareness around dynamic elements and the sharing of information with relevant key members. Future directions could include incorporating a more in depth focus on these elements in team training and measuring the impact of such training on team situational awareness with validated measures [24]. The development of cognitive aids has been previously promoted and needs to take into consideration team resuscitation communication patterns and teamwork principles [25]. Furthermore, such aids should be rigorously tested to ensure team performance is enhanced rather than degraded.

### Limitations

While this is the first mixed methods study to evaluate adult resuscitation team communication, it is not without limitations. Overall, this study was conducted at a single center and thus may only be generalizable to other urban academic tertiary centers but this is yet to be confirmed. For the interviews, since we relied on volunteers there is risk of self-selection bias, social desirability bias and recall bias. For the simulation observations, there were residents of varied levels of training which may have influenced the flow and sequence of events. In addition, there was the Hawthorne effect, and a risk that the fidelity of the simulation impaired authentic team communication. For the live observations, there was risk of data loss with the rapidity of case evolution and complexity of team communication and we attempted to verify this with the written health record. Video recording would have been helpful but would have required informed consent at our institution which is very difficult to obtain for emergency resuscitations. There is also risk of selection bias with the convenience sample we used for the live observations.

## Conclusions

Our findings across all three methods support a shared mental model among resuscitation team members. We identified clear information needs for resuscitation team members as well as a prioritized desire for team situational awareness. We observed consistent communication patterns whereby teams conveyed dense amounts of data in short periods of time. This study supports a need for cognitive aids to enhance teamwork and the acquisition and maintenance of situational awareness during resuscitations.

## Additional file

Additional file 1:
**Appendix A.** Questionnaire for stakeholder interviews. **Appendix B.** Communication categories observed during live resuscitation observations. **Appendix C.** Types of information exchanges observed during live resuscitations. **Appendix D.** Examples of team communication in live resuscitation observations. (DOC 160 kb)
