# Supplementary material for: Team communication patterns in emergency resuscitation: a mixed methods qualitative analysis
Source: Int J Emerg Med. 2017 Jul 14;10:24. doi: 10.1186/s12245-017-0149-4 (PMC5509566; doi:10.1186/s12245-017-0149-4)
Supplement: Additional file 1: — Appendix A. Questionnaire for stakeholder interviews. Appendix B. Communication categories observed during live resuscitation observations. Appendix C. Types of information exchanges observed during live resuscitations. Appendix D. Examples of team communication in live resuscitation observations. (DOC 160 kb) [file 12245_2017_149_MOESM1_ESM.doc]

**Additional file 1**

**Appendix A: Questionnaire for stakeholder interviews**

Questions:

1. What is your official role?
2. What is your responsibility in the team?
3. What is the team’s goal as a whole?
4. When was the last time you participated in a resuscitation scenario? (Trauma/non-trauma)
5. What are the types of scenarios that you may encounter?
6. What are the different types of trauma?
7. Who are the members of the team? (stake-holders involved in each of the scenario types)
8. What is the goal of each member of the team?
9. Choose one scenario and take us through the steps you would take, including: the typical sequence of each scenario and the exceptions, where the team is and what the physical position is of each team member.
10. Give more details about the sequence of scenarios.
11. What devices would you need and where are the artifacts and devices?
12. Talk about your role and your interaction with others.
13. What kind of information would you receive from others in the team?  How do you receive the information?
14. What kind of information is recorded? How? By whom?
15. Who does what, when, and where during the sequence of the scenario?
16. What is the most important info you need to get from someone? Who is that person?
17. Would you need devices that may not be located in the room?  How would you acquire a device that is not in the room?
18. How would you involve others who are not present in the room?  What kind of information do you need to pass on to those individuals?  Who sends them the information? How?
19. Are there any non-verbal interaction between the team members?
20. How long could each scenario take?
21. How do you keep time?  Are there any time limits for any particular procedure?
22. What information do you need to pass on to the next stage when the patient is moved from the resuscitation area?  Who transfers the information and to whom?
23. Talk about a situation that you may have not received vital information.

**Appendix B: Communication categories observed during live resuscitation observations (n=12)**


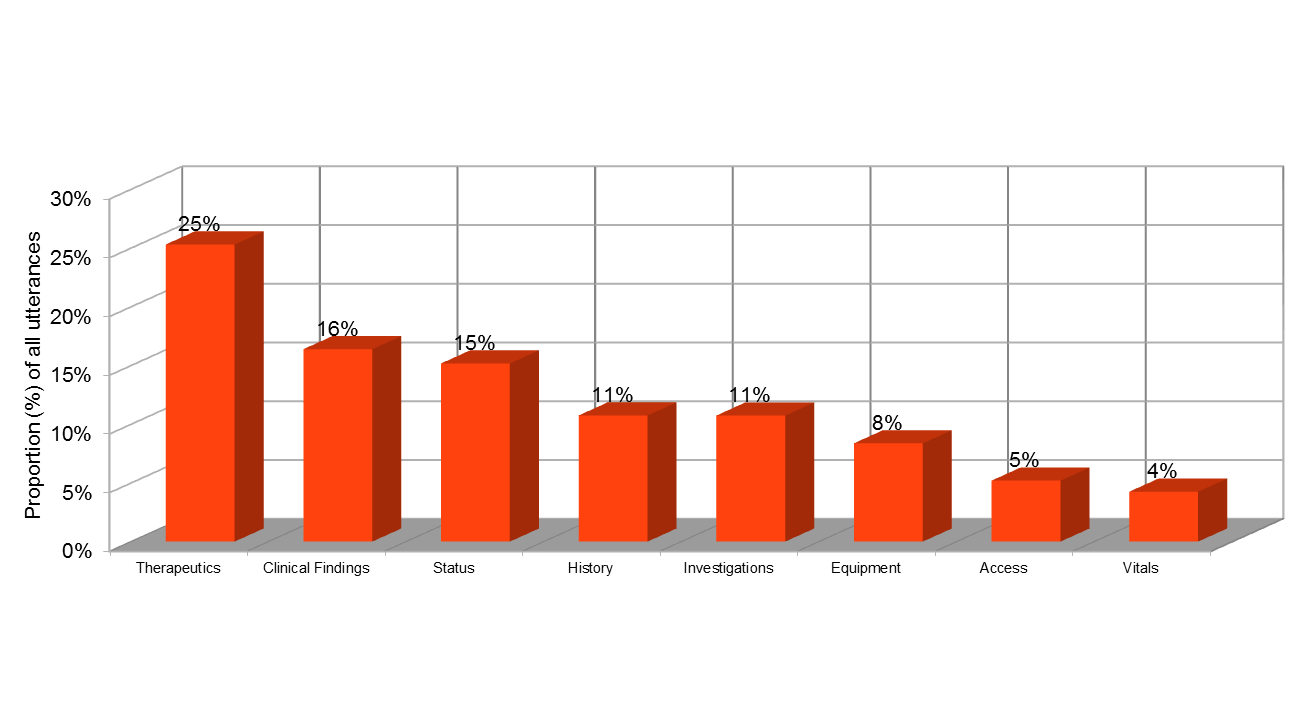


**Appendix C: Types of information exchanges observed during live resuscitations n=12**


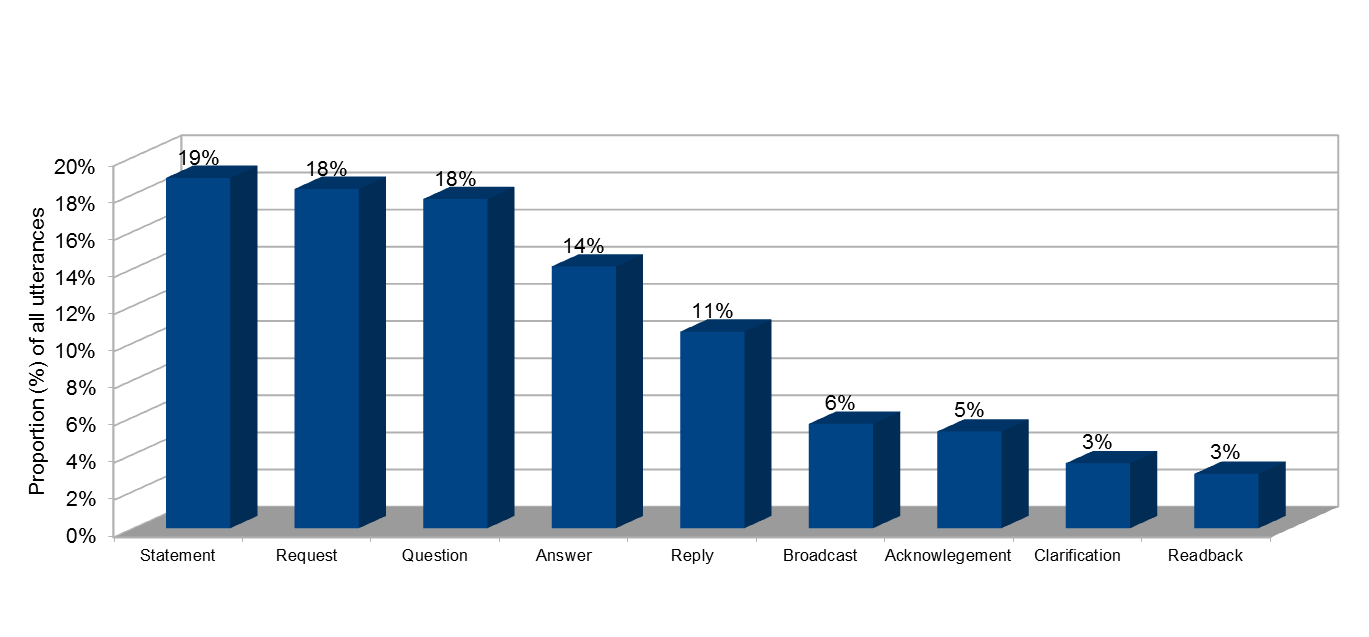


**Appendix D: Examples of team communication in live resuscitation observations**

|  | **Team Member Initiating Communication** | **Team Member Receiving Communication** | **Quotation** | **Type of Information Exchange** |
| --- | --- | --- | --- | --- |
| **Content Category Examples** | | | | |
| **Therapeutics** | MRP | RN2 | “Ready with the Ketamine?” | Question |
|  | RN2 | MRP | “Yeah I have it here” | Answer |
|  | MRP | RN3 | “And let's get a dopamine infusion ready” | Request |
| **Status** | RNch | MRP | “Two minutes since last shock, one since last epi” | Broadcast |
|  | MRP | RNch | “Two minutes?” | Question |
| **Clinical Findings** | MRP | Res1 | “What do you think of his GCS?” | Question |
|  | Res1 | MRP | “About a 7. Sort of localizing to pain, eyes are 3, verbal 2” | Answer |
|  | Res1 | MRP | “Resp rate 50's, lungs are very coarse” | Statement |
| **Verbal Behavior Examples** | | | | |
| **Statement** | MRP | RN1 | “Ok looks like we have a rhythm. No pulse though. Continue CPR.” | Status |
| **Request / Reply** | Res1 | RNch | “Has anyone given Mag?” | Therapeutics |
|  | RN1 | Res1 | “You want mag?” | Therapeutics |
|  | Res1 | RN1 | “Yes 2 of mag please.” | Therapeutics |
|  | RN1 | RNch | “Mag 2g given” | Therapeutics |
| **Question / Answer** | MRP | RNch | “Where are we on timing?” | Status |
|  | RNch | MRP | “15 mins since he arrived” | Status |

MRP= most responsible physician; RN=registered nurse, RNch=charting nurse, Res1=resident
